# Reproducibility of linear and angular cephalometric measurements obtained by an artificial-intelligence assisted software (WebCeph) in comparison with digital software (AutoCEPH) and manual tracing method

**DOI:** 10.1590/2177-6709.28.1.e2321214.oar

**Published:** 2023-04-03

**Authors:** S Tsander Tito Prince, Dilip Srinivasan, Sangeetha Duraisamy, Ravi Kannan, Krishnaraj Rajaram

**Affiliations:** 1 SRM Dental College, Department of Orthodontics and Dentofacial Orthopedics (Chennai, Tamil Nadu, India).

**Keywords:** Cephalometry, Artificial intelligence, Orthodontics

## Abstract

**Introduction:**

It has been suggested that human errors during manual tracing of linear/angular cephalometric parameters can be eliminated by using computer-aided analysis. The landmarks, however, are located manually and the computer system completes the analysis. With the advent of Artificial Intelligence in the field of Dentistry, automatic location of the landmarks has become a promising tool in digital Orthodontics.

**Methods:**

Fifty pretreatment lateral cephalograms obtained from the Orthodontic department of SRM dental college (India) were used. Analysis were done by the same investigator using the following methods: WebCeph™, AutoCEPH^©^ for Windows or manual tracing. Landmark identification was carried out automatically by Artificial Intelligence in WebCeph™ and with a mouse driven cursor in AutoCEPH^©^, and manually using acetate sheet and 0.3-mm pencil, ruler and a protractor. The mean differences of the cephalometric parameters obtained between the three methods were calculated using ANOVA with statistical significance set at p<0.05. Intraclass correlation coefficient (ICC) was used to determine both reproducibility and agreement between linear and angular measurements obtained from the three methods and intrarater reliability of repeated measurements. ICC value of >0.75 indicated good agreement.

**Results:**

Intraclass correlation coefficient between the three groups was >0.830, showing good level of agreement, and the value within each group was >0.950, indicating high intrarater reliability.

**Conclusion:**

Artificial Intelligence assisted software showed good agreement with AutoCEPH^©^ and manual tracing for all the cephalometric measurements.

## RESUMO

**Introdução:**

Tem sido sugerido que os erros humanos durante o traçado manual das medidas cefalométricas lineares/angulares podem ser eliminados usando a análise guiada por computador. Os pontos de referência, no entanto, são localizados manualmente e o sistema computadorizado completa a análise. Com o advento da Inteligência Artificial na Odontologia, a localização automática dos pontos de referência tornou-se uma ferramenta promissora na Ortodontia digital.

**Métodos:**

Cinquenta cefalogramas laterais pré-tratamento obtidos do departamento de Ortodontia da Faculdade de Odontologia SRM (Índia) foram usados. Todas as análises foram feitas pelo mesmo investigador, usando os seguintes métodos: WebCeph™, AutoCEPH^©^ para Windows ou traçado manual. A identificação dos pontos foi realizada automaticamente por Inteligência Artificial no WebCeph™; com o cursor do mouse, no AutoCEPH^©^; e manualmente, utilizando folha de acetato e lápis 0,3 mm, régua e transferidor. As diferenças médias dos parâmetros cefalométricos entre os três métodos foram calculadas por ANOVA com significância estatística fixada em p<0,05. O coeficiente de correlação intraclasse (ICC) foi usado para determinar a reprodutibilidade e a concordância entre as medidas lineares e angulares obtidas pelos três métodos e a confiabilidade intraexaminador para medidas repetidas. O valor de ICC > 0,75 indicou boa concordância.

**Resultados:**

O ICC entre os três grupos foi >0,830, mostrando bom nível de concordância, e o valor dentro de cada grupo foi >0,950, indicando alta confiabilidade intra-avaliador.

**Conclusão:**

O *software* assistido por Inteligência Artificial mostrou boa concordância com o AutoCEPH^©^ e o traçado manual para todas as medidas cefalométricas.

## INTRODUCTION

In the field of Orthodontics, cephalometric radiography is an essential tool for the treatment planning of underlying dental and skeletal discrepancies.^1^ It is also a valuable tool to evaluate treatment outcome and research. Conventional/manual analysis involves tracing of anatomic landmarks on an acetate sheet and measurement of the cephalometric parameters. The technique is time-consuming despite the wide-spread use in Orthodontics, and is largely dependent on the skills and knowledge of the clinician. In this context, errors in landmark identification due to fatigue may occur.^2^ ^,^ ^3^

Recently, cephalometric analysis using digitized software has gained attention and minimized many manual tracing related flaws. Another benefit is the possibility of conducting several analyses in a very short period of time, greatly minimizing human error due to fatigue.^4^ ^-^ ^6^ Other advantages of digitally acquired cephalometric imaging can be mentioned, such as a better recognition of the landmarks, image amplification and efficient storage of data. The future scope of using digital imaging in orthodontics is to make teleradiology a reality.^7^ ^,^ ^8^

Research conducted on digital cephalometry has found that the differences between the measurements derived from the digitally located landmarks and the conventional cephalometric radiographs were clinically acceptable, yet the results were found to be statistically significant. Different studies have evaluated the replicability of angular and linear measurements by various digital cephalometric computer programs such as Vistadent, Dolphin, and Quick Ceph.^9^ ^-^ ^13^

A two-dimensional (2D) artificial intelligence driven cephalometric program named ”WebCeph™” was programmed and made available as a web based platform for computers and also as a phone application. The most unique feature of WebCeph^TM^ is that it automatically identifies the landmarks using AI (artificial intelligence). 

Artificial intelligence can be a useful tool to reduce the time necessary for the final diagnosis and treatment planning.

As errors may occur during landmark identification, it is necessary to verify whether this AI-based software is reliable and reproducible when compared to a previously validated digital software (AutoCEPH^©^) and the traditional manual tracing.^14^

This study tests the null hypothesis that both linear and angular measurements acquired from two digitalized cephalometric analysis softwares (WebCeph™ and AutoCEPH^©^), as well as conventional method of tracing would not disagree to a statistically significant level.

## MATERIAL AND METHODS

### SAMPLE AND STANDARDIZATION

Fifty pretreatment lateral cephalograms were selected from patients treated at the SRM dental college, Ramapuram. Digital X-Ray machine (Villa System Rotograph, Villa Sistemi Medicali designs, Buccinasco, Italy) was used for taking the cephalograms of the patients using default settings: 72kVp and 06 mA with exposure at 4.50 seconds. Subjects were positioned at natural head position, teeth in centric occlusion with Frankfurt plane parallel to floor. Poor quality images or artifacts that could interfere with anatomical landmark identification were excluded.

For conventional method of tracing, no changes in resolution, contrast or brightness were made before printing. The cephalograms were printed on 8 x 10-in size radiographic film using (Drypix, Fujifilm,Tokyo, Japan) a compatible X-ray printer.

Based on the quantification of the known distance (e.g. 10 mm) between the two fixed points of the ruler present on the cephalostat of the digital x-ray system and on the digital images on the frame, adjustment of the true size of each cephalograph (in millimeters) was carried out.

### LANDMARKS IDENTIFICATION AND CEPHALOMETRIC PARAMETERS

**Figure 1:**
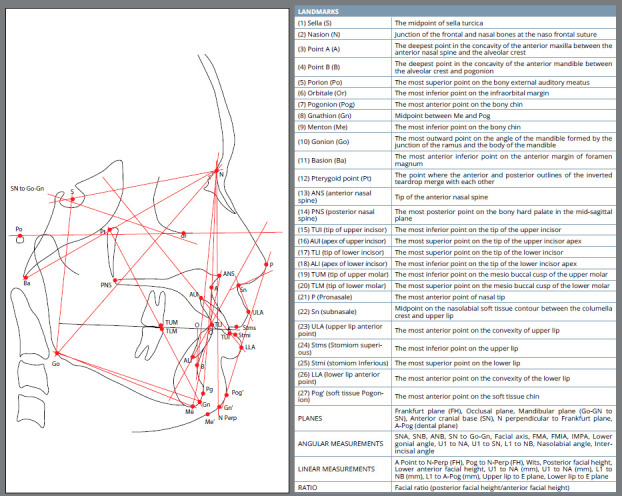
Commonly used anatomical landmarks and planes along with angular, linear parameters and ratio included in the study.

Twenty seven anatomical landmarks were marked on a cephalogram by the same investigator to evaluate commonly used cephalometric parameters used by orthodontists. The landmarks used in the study are described in (Fig 1).^1^ ^,^ ^15^ ^,^ ^16^

Subsequently, 25 cephalometric parameters were constructed from the 27 commonly used anatomical landmarks, comprising 10 linear , 14 angular parameters and 1 ratio. The measurements represented 13 skeletal, 9 dental, and 3 soft tissue related parameters (Table 1 and Fig 1). All linear and angular measurements of the conventional radiographs were recorded using a 0.3mm mechanical lead pencil on an acetate paper using a millimeter ruler and protractor. The obtained values were rounded off to 0.5mm or 0.5° respectively. Bilateral anatomical structures/landmarks were traced to an average single structure landmark. 

**Table 1:** Skeletal, dental and soft tissue parameters used in the study

SKELETAL PARAMETERS	DENTAL PARAMETERS	SOFT TISSUE
ANB	U1 to NA (degrees)	Nasolabial angle
SNA	U1 to SN (degrees)	Upper lip to E-plane
SNB	U1 to NA (mm)	Lower lip to E-Plane
A point to N-Perp (FH)	L1 to NB (mm)	
Pogonion to N-Perp (FH)	L1 to NB (degrees)	
Wits appraisal	L1 to A-Pog (mm)	
Mandibular plane angle (Go-Gn to SN)	FMIA	
Posterior facial height	IMPA	
Facial axis	Interincisal angle	
Facial height ratio (PFH/AFH)		
Lower anterior facial height		
FMA		
Lower gonial angle		

Landmark identification for AutoCEPH^©^ was carried out using a mouse-controlled cursor. For the WebCeph™, the landmarks were automatically identified and digitized by AI. After landmark identification the analysis of the various parameters were generated by both the softwares. 

Three readings were measured out and the average value was recorded. Excel spreadsheet was used to record the final readings.

To minimize errors due to human fatigue, only 5 cephalograms were analyzed per day both manually and digitally. 

Finally, 10 radiographs were randomly selected from the fifty radiographs and manually and digitally retraced, with a 10-day interval between assessments to test intra-observer reliability for analog and digital methods.

### STATISTICAL ANALYSIS

Statistical analysis was carried out using software version 26 of the Statistical Package for Social Sciences (SPSS Inc., IBM, Chicago, Illinois, United States). 

The cephalometric measurements of each parameter obtained from all the three tracing methods are presented as mean and standard deviation (Table 2). ANOVA (Analysis of variances) was used to verify any significant difference of cephalometric parameters obtained by the three tracing methods. Data distribution was normal in each group.^1^ Bonferroni analysis was use ad hoc. The level of significance was set at *p* < 0.05.

**Table 2:** Mean and standard deviation of cephalometric parameters obtained from manual tracing, AutoCEPH and WebCeph and the corresponding anova comparing the mean significance.

Variables	Mean values with standard deviation of cephalometric parameters			ANOVA
	MANUAL	AUTOCEPH	WEBCEPH	Sig.
SNA	80.94±4.33	81.36±4.65	81.18±4.22	0.892
SNB	78.44±4.63	78.48±5	78.42±4.75	0.998
ANB	2.72±3.53	2.94±3.27	2.74±2.94	0.933
FMA	26.66±7.4	26.6±7	23±7.22	0.101
FMIA	53.06±9.04	53.20±9.99	54.66±7.88	0.618
IMPA	99.18±11.58	9.18±10.91	100.64±8.59	0.722
MANDIBULAR PLANE ANGLE (GO-GN TO SN)	29.60±7.39	29.06±7.39	28.46±6.68	0.729
U1 to NA (Degrees)	33.12±8.32	32.98±8.12	30.90±80	0.315
U1 to SN	114.78±8.24	114.94±8.68	112.78±8.13	0.357
L1 to NB (Degrees)	30.72±8.64	30.68±9.78	29.4±7.42	0.688
INTERINCISAL ANGLE	111.68±12.87	111.98±13.72	115.80±11.35	0.198
FACIAL AXIS	87.86±5.15	89.86±5.69	88.66±4.83	0.162
LOWER GONIAL ANGLE	70.46±6.665	71.50±7.22	70.54±6.62	0.700
NASOLABIAL ANGLE	91.84±12.55	94±12.23	88±13.43	0.062
A to N-Perp (FH)	-3.360±4.5255	-2.710 ± 3.9150	-2.240 ±3.5488	0.827
Pog to N-Perp (FH)	-9.38±8.18	-8.75±7.64	-8.30±7.33	0.782
WITS APPRAISAL	1.60±3.68	1.76±3.73	1.40±2.95	0.874
FACIAL HEIGHT RATIO (PFH/AFH)	65.83±5.48	66.06±5.75	68.04±5.61	0.101
LOWER ANTERIOR FACIAL HEIGHT	67.84±7.13	68.24±7.35	65.76±6.64	0.172
U1 to NA (mm)	9.56±3.50	8.66±3.42	8.08±3.15	0.089
L1 to NB (mm)	7.8±3.76	7.04±3.74	6.98±3.09	0.438
L1 to A-Pog (mm)	5.7±4.10	4.44±3.84	4.46±3.80	0.187
UPPER LIP TO E-PLANE	-0.68±2.33	-0.92±2.69	-0.22±2.62	0.382
LOWER LIP TO E-PLANE	2.56±3.04	2.34±3.03	2.20±2.74	0.827
POSTERIOR FACIAL HEIGHT	77.64±7.86	78.44±7.65	78.08±7.01	0.868

Reproducibility of each cephalometric parameter was evaluated with the ICC by assessing the agreement between the values derived from WebCeph™, AutoCEPH and manual tracing. ICC value ≤ 0.75 indicated low agreement and a value > 0.75 indicated good agreement (Table 3). For the randomly selected 10 retraced radiographs, to assess the intrarater reliability for each tracing technique, the intraclass correlation coefficient (ICC) of the repeated cephalometric measurements was evaluated for 25 cephalometric parameters (Table 4).

**Table 3:** Intraclass correlation coefficients (ICCs) of cephalometric parameters obtained from manual tracing, AutoCEPH and WebCeph for assessing reproducibility.

VARIABLES	WEBCEPH VS AUTOCEPH VS MANUAL TRACING
	ICC
SNA	0.971
SNB	0.974
ANB	0.901
FMA	0.886
FMIA	0.910
IMPA	0.957
MANDIBULAR PLANE ANGLE (GO-GN TO SN)	0.932
U1 to NA (Degrees)	0.968
U1 to SN	0.968
L1 to NB (Degrees)	0.884
INTERINCISAL ANGLE	0.954
FACIAL AXIS	0.901
LOWER GONIAL ANGLE	0.870
NASOLABIAL ANGLE	0.893
A to N-Perp (FH)	0.830
Pog to N-Perp (FH)	0.942
WITS APPRAISAL	0.864
FACIAL HEIGHT RATIO (PFH/AFH)	0.914
LOWER ANTERIOR FACIAL HEIGHT	0.831
U1 to NA (mm)	0.890
L1 to NB (mm)	0.973
L1 to A-Pog (mm)	0.983
UPPER LIP TO E-PLANE	0.880
LOWER LIP TO E-PLANE	0.968
POSTERIOR FACIAL HEIGHT	0.929

## RESULTS

The mean and standard deviation of each cephalometric parameter obtained from the final readings were tabulated and subjected to analysis by ANOVA, indicating no statistically significant difference between the cephalometric measurements among the three methods at p<0.05 (Table 2). The Intraclass Correlation Coefficient between the three methods showed that all the parameters had values from 0.830-0.983 indicating high level of agreement among the three tracing methods. The highest ICC value was for L1 to A-Pog(mm) (Table 3). All ICC values of repeated measurements within each group obtained have shown more than 0.950, indicating very high intrarater reliability (Table 4).^17^ ^,^ ^18^

**Table 4:** Intraclass correlation coefficients (ICCs) of repeated cephalometric measurements obatined from WebCeph, AutoCEPH and manual method for assessing intra-rater reliability.

VARIABLES	ICC FOR WEBCEPH	ICC FOR AUTOCEPH	ICC FOR MANUAL TRACING
SNA	0.984	0.998	0.992
SNB	0.976	0.978	0.988
ANB	0.970	0.986	0.985
FMA	0.980	0.988	0.995
FMIA	0.974	0.994	0.978
IMPA	0.995	0.995	0.998
MANDIBULAR PLANE ANGLE (GO-GN TO SN)	0.988	0.990	0.992
U1 to NA (Degrees)	0.989	0.972	0.950
U1 to SN	0.983	0.971	0.980
L1 to NB (Degrees)	0.997	0.988	0.994
INTERINCISAL ANGLE	0.978	0.978	0.950
FACIAL AXIS	0.984	0.986	0.950
LOWER GONIAL ANGLE	0.992	0.988	0.980
NASOLABIAL ANGLE	0.988	0.994	0.994
A to N-Perp (FH)	0.975	0.974	0.968
Pog to N-Perp (FH)	0.985	0.986	0.968
WITS APPRAISAL	0.978	0.950	0.957
FACIAL HEIGHT RATIO (PFH/AFH)	0.988	0.980	0.952
LOWER ANTERIOR FACIAL HEIGHT	0.976	0.994	0.957
U1 to NA	0.998	0.995	0.952
L1 to NB	0.974	0.988	0.988
L1 to A-Pog	0.985	0.989	0.985
UPPER LIP TO E-PLANE	0.980	0.983	0.995
LOWER LIP TO E-PLANE	0.992	0.994	0.978
POSTERIOR FACIAL HEIGHT	0.991	0.986	0.998

## DISCUSSION

In this study, the AI-based landmark digitization was tested and validated with commercially available digital software and manual tracing. The results have shown good reproducibility.^17^ ^,^ ^19^

Considering AutoCEPH^©^ an ideal tool for Indian population, this digital software was chosen for comparison with WebCeph™ and manual tracing.^14^ This study was therefore carried out in order to compare and evaluate the reproducibility of cephalometric analysis between the newly introduced AI web based orthodontic software versus the indigenously developed AutoCEPH^©^ newer version (1.1.3) along with the conventional method of tracing.

ANOVA indicated that there was no statistical significance difference between the three methods (Table 2). ICC showed high level of agreement (Table 3) for all the variables, indicating acceptable reproducibility of the cephalometric parameters of the WebCeph™ when compared with AutoCEPH^©^ and manual method of tracing. It is thus assumed that AI-based software can be used for cephalometric analyses. Based on the findings listed above it can be stated that the null hypothesis fails to be rejected, which is in agreement with a previous study.^17^

The explanation for relatively lower ICC value for A to N-Perp (FH) can be due to the fact that sometimes the landmarks Porion and Orbitale are not clearly identifiable, which has also been reported in previous studies.^1^ ^,^ ^13^ ^,^ ^17^ ^,^ ^18^ ^,^ ^20^

 Parameters such as Lower anterior facial height, FMA, L1 to NB (degrees), Lower Gonial angle, Nasolabial angle, Wits, U1 to NA (mm) and Upper lip to E-plane showed ICC value >0.83 but <0.90. These results might have occurred due to inconsistencies in defining the landmarks Go, Gn, N , Lower incisor apex and U1 to NA, as it has been repeatedly reported in previous studies.^12^ ^,^ ^21^ ^-^ ^23^

Soft tissue parameters such as nasolabial angle and upper lip to E plane may present differences between the digital softwares in locating the soft tissue borders of the lip (ULA, LLA, Sn, P and Pg’)^24^; however, both softwares incorporate features to relocate the points after initial digitization to minimize landmark error. Nevertheless, it is important to mention that a difference of less than two (degrees or millimeters) is considered to be within clinical acceptable limits.^11^ ^,^ ^25^

Following the indication of previous studies, only one operator was involved with all cephalometric measurements in this study, as intrarater examination error is far greater than inter examination error.^18^ ^,^ ^21^ Similarly, only the commonly used and easily locatable anatomical landmarks in cephalometric analyses were selected.^1^ ^,^ ^15^ ^,^ ^16^

Intra rater reliability of repeated measurements showed value of ≥ 0.950, indicating that the level of agreement of measurements obtained from the 1^st^ and the 2^nd^ repeated tracings in each method was reliable (Table 4). The findings from the intra rater statistics suggests that AI-assisted landmark identification is reliable and acceptable, which reinforces that WebCeph™ is reliable can be used as a routine cephalometric tool, hence supporting the study done by Hwang et al.^26^

Digital cephalometry provides many advantages in terms of fatigue and ease of application, however, the landmark identification process is operator dependent and in case of multiple cephalometric analysis can be tiring and time consuming.^1^ ^,^ ^2^ ^,^ ^5^ ^,^ ^6^ ^,^ ^17^ ^,^ ^18^ ^,^ ^20^ ^,^ ^24^ With the introduction of AI-based landmark identification software WebCeph™, the process of digitization has become easy and rapid. The main objective for incorporating AI in cephalometrics is to reduce the work load of orthodontists and allow easy access through an online portal for computers and mobile phone from anywhere in the world.^27^

AI-based digital softwares require high resolution lateral cephalogram and absence of structures superimposition, because of possible interferences with the algorithm for landmark identification.^26^ This disadvantage is not seen in manual tracing as the operator can differentiate and evaluate the structures based on sound knowledge and judgment. 

## LIMITATIONS

The ability to analyze landmark by AI is solely dependent on radiograph quality and resolution. It is also dependent on internet connection and cannot be accessed from remote areas where network is not available. AI cannot identify or approximate bilateral structures which are superimposed on the radiograph.

## FUTURE SCOPE

With the advent of teleradiology, the online based AI software WebCeph™ can be used for both teaching and training from traditional locations and also successfully improving the orthodontic referrals and expertise through technology. It is anticipated the compatibility with mobile devices and availability as a smartphone app. Further 3D based AI algorithms can be developed to construct and automatically identify landmarks and construct the various cephalometric analyses.

## CONCLUSION

The Artificial Intelligence software WebCeph™ showed high level of agreement in terms of reliability with earlier validated software AutoCEPH^©^ and manual tracing. The agreement of the softwares for the repeated measurements was found to be adequate, suggesting that it can be used for routine cephalometric analysis and clinical research by the orthodontists.

## References

[1] Celik E, Polat-Ozsoy O, Toygar Memikoglu TU (2009). Comparison of cephalometric measurements with digital versus conventional cephalometric analysis. Eur J Orthod.

[2] Baumrind S, Frantz RC (1971). The reliability of head film measurements 1. Landmark identification. Am J Orthod.

[3] Baumrind S, Frantz RC (1971). The reliability of head film measurements 2. Conventional angular and linear measures. Am J Orthod.

[4] Tsorovas G, Karsten AL-A (2010). A comparison of hand-tracing and cephalometric analysis computer programs with and without advanced features--accuracy and time demands. Eur J Orthod.

[5] Liu J-K, Chen Y-T, Cheng K-S (2000). Accuracy of computerized automatic identification of cephalometric landmarks. Am J Orthod Dentofacial Orthop.

[6] Erkan M, Gurel HG, Nur M, Demirel B (2012). Reliability of four different computerized cephalometric analysis programs. Eur J Orthod.

[7] Tan SS, Ahmad S, Moles DR, Cunningham SJ (2011). Picture archiving and communications systems a study of reliability of orthodontic cephalometric analysis. Eur J Orthod.

[8] Mandall NA, O'Brien KD, Brady J, Worthington HV, Harvey L (2005). Teledentistry for screening new patient orthodontic referrals Part 1: A randomised controlled trial. Br Dent J.

[9] Gregston MD, Kula T, Hardman P, Glaros A, Kula K (2004). A comparison of conventional and digital radiographic methods and cephalometric analysis software Hard tissue. Semin Orthod.

[10] Roden-Johnson D, English J, Gallerano R (2008). Comparison of hand-traced and computerized cephalograms landmark identification, measurement, and superimposition accuracy. Am J Orthod Dentofacial Orthop.

[11] Chen Y-J, Chen S-K, Chung-Chen Yao J, Chang H-F (2004). The effects of differences in landmark identification on the cephalometric measurements in traditional versus digitized cephalometry. Angle Orthod.

[12] Santoro M, Jarjoura K, Cangialosi TJ (2006). Accuracy of digital and analogue cephalometric measurements assessed with the sandwich technique. Am J Orthod Dentofacial Orthop.

[13] Uysal T, Baysal A, Yagci A (2009). Evaluation of speed, repeatability, and reproducibility of digital radiography with manual versus computer-assisted cephalometric analyses. Eur J Orthod.

[14] Mahto RK, Kharbanda OP, Duggal R, Sardana HK (2016). A comparison of cephalometric measurements obtained from two computerized cephalometric softwares with manual tracings. J Indian Orthod Soc.

[15] Forsyth DB, Davis DN (1996). Assessment of an automated cephalometric analysis system. Eur J Orthod.

[16] Tong W, Nugent ST, Gregson PH (1990). Jensen GM, Fay DF Landmarking of cephalograms using a microcomputer system. Comut Biomed Res.

[17] Goracci C, Ferrari M (2014). Reproducibility of measurements in tablet-assisted, PC-aided, and manual cephalometric analysis. Angle Orthod.

[18] Sayinsu K, Isik F, Trakyali G, Arun T (2007). An evaluation of the errors in cephalometric measurements on scanned cephalometric images and conventional tracings. Eur J Orthod.

[19] Forsyth DB, Shaw WC, Richmond S, Roberts CT (1996). Digital imaging of cephalometric radiographs, part 2 image quality. Angle Orthod.

[20] Bruntz LQ, Palomo JM, Baden S, Hans MG (2006). A comparison of scanned lateral cephalograms with corresponding original radiographs. Am J Orthod Dentofacial Orthop.

[21] Livas C, Delli K, Spijkervet FK, Vissink A, Dijkstra PU (2019). Concurrent validity and reliability of cephalometric analysis using smartphone apps and computer software. Angle Orthod.

[22] Polat-Ozsoy O, Gokcelik A, Toygar Memikoglu TU (2009). Differences in cephalometric measurements a comparison of digital versus hand-tracing methods. Eur J Orthod.

[23] Chen YJ, Chen SK, Chang HF, Chen KC (2000). Comparison of landmark identification in traditional versus computer-aided digital cephalometry. Angle Orthod.

[24] Kublashvili T, Kula K, Glaros A, Hardman P, Kula T (2004). A comparison of conventional and digital radiographic methods and cephalometric analysis software II. Soft tissue. Semin Orthod.

[25] Zamrik OM, Iseri H (2021). The reliability and reproducibility of an android cephalometric smartphone application in comparison with the conventional method. Angle Orthod.

[26] Hwang HW, Park JH, Moon JH, Yu Y, Kim H, Her SB (2020). Automated identification of cephalometric landmarks Part 2-Might it be better than human?. Angle Orthod.

[27] Kohli SS, Kohli VS (2020). Comparison of reproducibility of cephalometric measurements derived from handheld (smartphone) device application versus manual cephalometric tracing. Int J Orthod Rehabil.

[28] Shettigar P, Shetty S, Naik RD, Basavaraddi SM, Patil AK (2019). A comparative evaluation of reliability of an android-based app and computerized cephalometric tracing program for orthodontic cephalometric analysis. Biomed Pharmacol J.

